# Phosphoric acid containing proanthocyanidin enhances bond stability of resin/dentin interface

**DOI:** 10.1590/0103-6440202203941

**Published:** 2022-08-26

**Authors:** Yumi C. Del Rey, Regina G. Palma-Dibb, Rodrigo França, Francisco W.G. Paula-Silva, Débora F.C. Guedes, Cristina Fiuza, Ana C.B.C.J. Fernandes, Juliana J. Faraoni, Lourenço M.R. Roselino

**Affiliations:** 1 Departmento de Odontologia Restauradora, Faculdade de Odontologia de Ribeirão Preto, Universidade de São Paulo, Ribeirão Preto, SP, Brazil; 2 Departmento de Clínica Infantil, Faculdade de Odontologia de Ribeirão Preto, Universidade de São Paulo, Ribeirão Preto, SP, Brazil; 3 Department of Restorative Dentistry, Faculty of Dentistry, University of Manitoba, Winnipeg, MB, Canada; 4 Department of Oral Biology, Faculty of Dentistry, University of Manitoba, Winnipeg, MB, Canada.

**Keywords:** Dentin biomodification, matrix metalloproteinases, in situ zymography, microtensile bond strength, adhesive interface

## Abstract

Proanthocyanidin (PA) is a promising dentin biomodifier due to its ability to stabilize collagen fibrils against degradation by matrix metalloproteinases (MMPs); however, the most effective protocol to incorporate PA into bonding procedures is still unclear. This study evaluated the effect of dentin biomodification with a PA acid etchant on MMP activity, adhesive interface morphology and resin-dentin microtensile bond strength. Sound extracted human molars were flattened to expose dentin and acid-etched for 15 s according to the groups: EXP - experimental phosphoric acid; EXP+PA - experimental phosphoric acid 10% PA; TE - total-etching system; SE - self-etching system. Samples were restored with composite resin and stored in distilled water (37ºC). MMP activity and interface morphology were analyzed after 24 h by in situ zymography (n=6) and scanning electron microscopy (n=3), respectively. The resin-dentin microtensile bond strength (μTBS) was evaluated after 24 h and 6 months storage (n=6). Significantly higher MMP activity was detected in etched dentin compared with untreated dentin (p<0.05), but no difference among acid groups was found. Resin tags and microtags, indicative of proper adhesive system penetration in dentinal tubules and microtubules, were observed along the hybrid layer in all groups. There was no difference in μTBS between 24 h and 6 months for EXP+PA; moreover, it showed higher long-term μTBS compared with TE and EXP (p<0.05). The results suggest that 15 s of biomodification was not sufficient to significantly reduce MMP activity; nonetheless, EXP+PA was still able to improve resin-dentin bond stability compared with total- and self-etching commercial systems.

## Resumo

A proantocianidina (PA) é um biomodificador dentinário promissor devido a sua capacidade de estabilizar as fibrilas colágenas contra a degradação por metaloproteinases da matriz (MMPs); no entanto, o protocolo mais eficaz para a incorporação de PA em procedimentos adesivos ainda não está claro. Este estudo avaliou o efeito da biomodificação da dentina com um condicionador ácido contendo PA na atividade de MMPs, morfologia da interface adesiva e resistência à microtração resina-dentina. Molares humanos extraídos foram lixados para exposição da dentina e condicionados com ácido por 15 s de acordo com os grupos: EXP - ácido fosfórico experimental; EXP+PA - ácido fosfórico experimental com 10% PA; TE - sistema total-etch; SE - sistema self-etch. As amostras foram restauradas com resina composta e armazenadas em água destilada (37ºC). A atividade de MMP e morfologia da interface foram analisadas após 24 h por zimografia in situ (n=6) e microscopia eletrônica de varredura (n=3), respectivamente. A resistência à microtração resina-dentina (μTBS) foi avaliada após 24 horas e 6 meses de armazenamento (n=6). Atividade de MMP detectada na dentina condicionada foi significativamente maior em comparação com a dentina não tratada (p <0,05), mas não houve diferenças entre os diferentes ácidos. Tags e microtags de resina, indicativos de uma penetração adequada do sistema adesivo nos túbulos e microtúbulos dentinários, foram observadas ao longo da camada híbrida em todos os grupos. Não houve diferença entre os valores de μTBS de 24 h e 6 meses para EXP+PA; além disso, EXP+PA apresentou maiores valores de μTBS após 6 meses em comparação com TE e EXP (p <0,05). Os resultados sugerem que a biomodificação por 15 s não foi suficiente para reduzir significativamente a atividade de MMP; apesar disso, EXP + PA foi capaz de melhorar a estabilidade da interface resina-dentina em comparação com sistemas total- e self-etch comerciais.

## Introduction

A major challenge in restorative dentistry is to overcome deficiencies of current adhesive systems and improve the clinical longevity of resin composite restorations ^1^. Once exposed to the oral environment, the resin-dentin interface slowly undergoes hydrolysis of its hydrophilic resinous components by esterases, and degeneration of exposed collagen fibrils by matrix metalloproteinases (MMPs) and cathepsins, such as MMP-2, -8 and -9 ^2^. MMPs are enzymes present in the organic portion of dentin capable of cleaving collagen fibrils that are not protected by hydroxyapatite or resin. During secretion of dentin matrix, MMPs are produced by odontoblasts and remain trapped within the calcified matrix in an inactive form until caries, erosion and/or acid etching for adhesive restoration releases and activates them ^1^ ^,^ ^2^. Within time, this progressive interface degradation can lead to interfacial nanoleakage, loss of adhesive bond strength and compromised longevity of resin composite restorations ^(^ ^1^ ^,^ ^2^ ^,^ ^3^.

Different strategies can be used for bonding to dentin regarding the acidity of the etchants. Total-etching adhesive systems involve the application of a strong phosphoric acid (35-37%) that completely removes the smear layer and exposes the collagen network in a depth of 3-7 µm ^3^. The primer and adhesive applied in sequence infiltrate the interfibrillar spaces and form a hybrid layer that protects the collagen fibrils from hydrolysis ^1^ ^,^ ^2^. However, the demineralized dentin is not fully infiltrated by resin monomers, and fibrils at the bottom of the hybrid layer remain exposed and susceptible to degradation by MMPs released and activated by the etching procedures ^4^. On the other hand, self-etching systems rely on acidic monomers of the primer to etch the dentin surface and partially remove the smear layer, incorporating it to the hybrid layer ^2^ ^,^ ^3^. Since the dentin demineralization and the resin monomers infiltration occur at the same time, theoretically this protocol does not create a region of unprotected fibrils ^2^. Nonetheless, it has been demonstrated that small areas of incomplete monomer infiltration can be observed even when using self-etching systems, and that further degradation of the hydrophilic resin components also leads to areas of exposed collagen susceptible to hydrolysis ^2^ ^,^ ^3^.

In this context, several studies have investigated the use of collagen cross-linker agents (*e.g.* glutaraldehyde, carbodiimide, riboflavin, chlorhexidine and proanthocyanidin) as dentin biomodifiers ^5^ ^,^ ^6^. Cross-linkers as substances capable of forming new links between the collagen chains, known as cross-links, which enhance the mechanical properties of the collagen fibrils against proteolytic degradation ^6^. Other studies have also demonstrated that cross-linkers are able to minimize dentin MMP activity ^6^ ^,^ ^7^. Among those, proanthocyanidin (PA) is a natural substance that can be easily obtained from plant sources such as grape seeds, cocoa seeds, cinnamon and green tea extract ^7^. Compared with glutaraldehyde, riboflavin and chlorhexidine, PA has demonstrated to be the most effective in reducing MMP activity ^6^ ^,^ ^7^. Moreover, PA does not affect cell viability and proliferation, which makes its use safe in dentistry and advantageous over certain synthetic cross-linkers that present high cytotoxicity, such as glutaraldehyde ^6^. Therefore, PA is a promising dentin biomodifier to increase the longevity of adhesive restorations.

A fundamental aspect to the viability of the use of cross-linker agents is a clinically short biomodification time ^8^. Several studies used considerably long biomodification protocols ^9^ ^,^ ^10^ ^,^ ^11^ ^,^ ^12^ or added it as an extra step for resin composite restorations ^4^ ^,^ ^13^. In this context, it is still unclear what is the most effective and timesaving protocol for PA incorporation into bonding procedures. Therefore, the aim of the present study was to evaluated the effect of dentin biomodification for 15 s with an experimental phosphoric acid containing 10% PA on MMP activity, adhesive interface morphology and resin-dentin microtensile bond strength. The null hypothesis was that the application of an experimental etchant containing PA would not influence the analyzed parameters when compared to an experimental etchant without PA and commercial total- and self-etching systems. 

## Materials and methods

The present study was approved by the Ethics Committee of the School of Dentistry of Ribeirão Preto at University of São Paulo (CAAE 68497217.0.0000.5419).

### Experimental solutions

Phosphoric acid (85 wt.% in H2O), obtained from Sigma-Aldrich (Milwaukee, WI, USA), was diluted in a 50/50% water-propylene glycol solution to obtain a 35% experimental phosphoric acid. A thickening agent was added to increase the solution viscosity and make it more resistant to flow, avoiding the etching of undesirable spots. In order to produce an experimental phosphoric acid containing PA, grape seed extract (GSE, Vitis vinifera, PA≥95%, Farmácia de Manipulação Luva Ervas, Caieiras, SP, Brazil) was dissolved in the 35% experimental acid solution prior to the thickening step, to a final concentration of 10% w/v of PA. The resulting mixture underwent magnetic stirring for 24 h under room temperature until complete dissolution of the GSE, followed by addition of the thickening agent. The final solutions were stored at 4ºC. 

### Specimen preparation and bonding procedures

Twenty-four sound extracted human molars had their occlusal enamel and roots removed perpendicularly to their long axis using a diamond disc attached to a cutting machine (Minitrom: Struers A/S, Copenhagen, Denmark) at 350 RPM under constant water cooling. The roots were sectioned 2 mm below the cementoenamel junction and all pulp tissue remnants were removed. The pulp chamber was restored using adhesive system (Adper Scotchbond MP, 3M ESPE, St. Paul, MN, USA) and Filtek Z350 (3M ESPE) resin composite, applied according to the manufacturer’s instruction (Box 1). Subsequently, the occlusal side was ground flat using #280 - #600 grit silicon carbide papers under running water (Politriz Arotec APL- 4, Arotec S/A Ind. e Comércio, São Paulo, SP, Brazil) in order to fully expose the coronal dentin and standardize the smear layer. The teeth were then randomly treated according to the following groups (n=6): EXP - 35% experimental phosphoric acid; EXP+PA - 35% experimental phosphoric acid containing 10% PA; TE - total-etching system (Ultra-Etch, Indaiatuba, SP, Brazil) and SE - self-etching system (Clearfil SE Bond, Noritake Dental Inc., Osaka, Japan). TE was included as a control group since the experimental acids are also classified as total-etching systems. SE was used as a second control because it is the current gold standard for adhesion to dentin ^2^. For EXP+PA, EXP and TE, the acid treatments were applied for 15 s in dentin, the samples were rinsed, dried and the adhesive system (Adper Scotchbond MP, 3M ESPE) was applied following the manufacturer’s instruction. SE samples were treated using the acid primer and bond components of Clearfil SE Bond (Noritake Dental Inc.) self-etching system. The detailed description of materials, manufacturers and application protocols are presented in Box 1. All groups were restored using 1 mm layers of resin composite (Filtek Z350, 3M ESPE) light-cured for 20 s using a LED light-curing unit (DB 685, Dabi Atlante, Ribeirão Preto, São Paulo, Brazil) with an irradiance of 700 mW/cm². The restored specimens were then longitudinally sectioned in mesial-distal and buccal-lingual directions to produce 0.8 x 0.8 mm beams using a diamond saw under constant water cooling in a cutting machine (Minitrom: Struers A/S). The beams were stored at 37ºC in distilled water, which was replaced weekly for fresh amounts, and used to perform *in situ* zymography and microtensile bond tests.

**Box 1:**
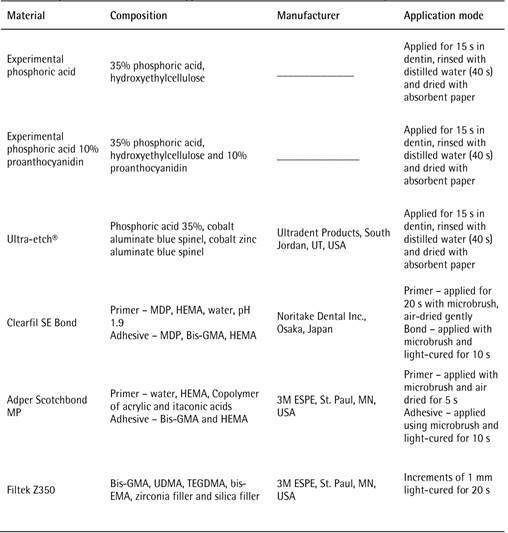
Composition, manufacturer and application mode of materials used in the study.

Bis-GMA - bisphenol A-glycidyl methacrylate; HEMA - hydroxyethylmethacrylate; MDP - 10-methacryloyloxy decyldihydrogenphosphate; UDMA - urethane dimethacrylate; bis-EMA - ethoxylated bisphenol A glycol dimethacrylate; TEGDMA - triethylene glycol dimethacrylate.

### Microtensile Bond Test

The microtensile bond test was performed after 24 h and 6 months of storage using 5 beams per tooth (n=6). The microtensile bond strength values for all beams from the same tooth were averaged and each tooth was considered as the statistical unit. The beams were fixed to a jig using cyanoacrylate glue (Superbonder, Gel-Henkel Loctite Adesivos Ltda., São Paulo, SP, Brazil), placed on an Universal Testing Machine (DL 2000, EMIC Equipamentos e Sistemas de Ensaio Ltda., São José dos Pinhais, PR, Brazil) and subjected to tensile forces at a crosshead speed of 0.5mm/min, with 500 load cell, until debonding. Microtensile bond strength values (MPa) were calculated by dividing the peak force (N) by the area of bonding (mm²) measured using a digital caliper. The broken beams were examined under confocal laser scanning microscopy (OLS 4000, Carl Zeiss, Oberkochen, Germany) at 10x magnification to determine the failure mode. Fractures were classified as adhesive (failure at the resin-dentin interface), cohesive (failure within dentin or resin portion) or mixed (adhesive and cohesive). The percentages of the fracture modes were recorded for all groups at the two experimental periods.

### *In situ* zymography 

The MMP activity at the adhesive interface was determined by *in situ* zymography, performed after 24 h of storage. A control group consisting of beams that did not receive acid or adhesive treatment before restoration was used to measure the basal fluorescence activity of dentin. Beams (n=6) were immersed for 15 min (3x) in a 1.0 mg/mL borohydride sodium solution (Sigma Corporation, Tokyo, Japan) and rinsed with phosphate-buffered saline (PBS). Subsequently, a fluorescein-conjugated gelatin substrate (DQ™ Gelatin, Molecular Probes, Eugene, OR, USA) dissolved in PBS to a 1mg/mL concentration was used to incubate the specimens for 3 h at 37°C in a humidified dark chamber. In order to verify if the observed proteolytic activity was due to MMP enzymes, additional slices were pre-incubated in 20 mM ethylenediaminetetraacetic acid (EDTA, Sigma Corporation), a strong MMP inhibitor, for 1 h and then immersed in the gelatinous substrate. The hydrolysis of the fluorescein-conjugated gelatin substrate, indicative of MMP activity, was evaluated under a fluorescence microscope at 100x magnification (10x objective lens) using the Alexa Fluor 43HE filter (FT 570, BP 550/25, BP 605/70, Carl Zeiss). The fluorescence emission was analyzed by densitometry using ImageJ software (National Institutes of Health, Bethesda, MD, USA) and expressed as arbitrary units of fluorescence per mm^2^.

### Scanning Electron Microscopy (SEM)

Three sound extracted human molars had their roots cut off using a low-speed saw (IsoMet, Buehler Ltd., Evanston, IL, USA). The occlusal side was ground flat using number #180 grit silicon carbide paper under running water to expose the dentin surface. On each specimen, 4 cavities (1.5 mm depth, 4 mm buccolingual length and 1.5 mm mesiodistal width) were produced in dentin using a cylindrical diamond bur (Shofu Inc., Kyoto, Japan) in a high-speed handpiece under water cooling. The samples were then washed ultrasonically in distilled water (15 min) and each cavity was treated and restored according to the 4 abovementioned groups. Using a water-cooled diamond saw in a cutting machine (Minitrom, Struers A/S, Copenhagen, Denmark), the specimens were longitudinally cut in the mesiodistal direction to produce two slices each (one buccal and one lingual) with the hybrid layer, dentin and resin composite areas exposed. The resulting slices were individually fixed inside a stainless-steel ring with the hybrid layer facing up. Self-polymerizing acrylic resin (Epofix Harden, Struers A/S), manipulated according to the manufacturer’s instruction, was used to embed the samples without coating the surface. After the resin polymerization, the specimens were polished under water cooling using #600 - #2000 grit silicon carbide papers (1 min each) followed by felt discs with aluminum oxide pastes (1.0 and 0,5 µm) for 1 min each.

In order to enable a clear visualization of the hybrid layer and the resin tags, demineralization and deproteinization treatments were performed. The specimens were embedded in 85% phosphoric acid (Sigma-Aldrich, Milwaukee, WI, USA) for 3 min and then incubated for 10 min in 1% sodium hypochlorite solution (Sigma-Aldrich), rinsed with distilled water, air dried and stored at room temperature for 24 h. Afterwards, the specimens were sputter-coated with a layer of approximately 50 nm thickness of gold-palladium alloy at 50 militorr for 45 s (Desk II Cold Sputter Unit, Denton Vacuum LLC, Moorestown, NJ, USA). Images were obtained from the hybrid layer area at magnifications of 1350x, 3737x and 21600x using a high-resolution SEM (Quanta FEG 650; FEI, Hillsboro, OR, USA). 

### Statistical analysis

Data was submitted to a normality test (Shapiro-Wilk) and presented normal distribution. Statistical analysis was performed using One-Way ANOVA for the *in situ* zymography test (test power of 84.7%) and Two-Way Mixed Model ANOVA for the microtensile bond strength test (test power of 85.3% for the factor “treatment”, 91.1% for the factor “time” and 90.1% for “treatment x time” interaction). Multiple comparisons were performed using Tukey’s Test, with a significance level of α=0.05. All analyses were performed using the SPSS Software for Windows version 21.0 (SPSS Inc., Chicago, IL, USA) and GraphPad Prism 5.0 (GraphPad Software, Inc., San Diego, CA, USA).

## Results

### Microtensile Bond Test

**Figure 1:**
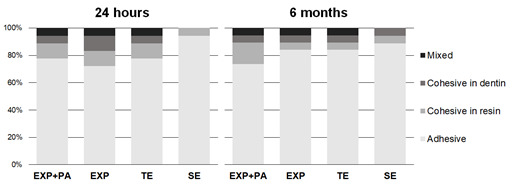
Fracture analysis of specimens submitted to microtensile bond strength test at 24 h and 6 months storage (%). EXP - experimental phosphoric acid; EXP+PA - experimental phosphoric acid 10% PA; TE - total-etching system and SE - self-etching system.

Microtensile bond strength means and standard deviations, according to the acid treatment and storage time, are shown in Table 1. At 24 h, EXP+PA showed no statistical difference compared with the commercial groups (TE and SE). No difference between immediate (24 h) and long-term (6 months) microtensile bond strengths was found for EXP+PA and SE, while values decreased significantly (*p*<0.05) for EXP and TE. After 6 months of storage, EXP+PA showed a statistically higher microtensile bond strength (*p<*0.05) compared with TE and EXP, and no statistical difference compared with SE. The fracture analysis showed predominantly adhesive failures in all groups at 24 h (~81%) and 6 months (~86%) (Figure 1).

**Table 1:** Microtensile bond strength means and standard deviations 24 h and 6 months after acid treatments.

Groups	24 hours (MPa)	6 months (MPa)
*EXP*	59.25 (10.32)aA	30.88 (8.93)bB
*EXP+PA*	47.10 (7.01)aB	41.10 (3.92)aA
*TE*	54.01 (6.97)aAB	29.47 (7.51)bB
*SE*	36.61 (6.77)aCB	35.46 (4.73)aAB

Equal letters mean no statistically significant difference (2-way mixed model ANOVA, Tukey’s Test, p<0.05). Small letters compare the two time periods for each acid treatment. Capital letters compare different acid treatments at the same time point. EXP - experimental phosphoric acid; EXP+PA - experimental phosphoric acid 10% PA; TE - total-etching system and SE - self-etching system.

### In situ Zymography

**Figure 2:**
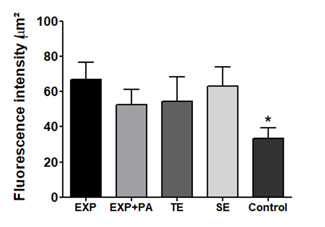
Fluorescence emission quantification of MMP activity detected by *in situ* zymography (1-way-ANOVA, Tukey’s Test, *p<0,05 for control compared to acid groups). EXP - experimental phosphoric acid; EXP+PA - experimental phosphoric acid 10% PA; TE - total-etching system; SE - self-etching system; Control: untreated dentin.

The *in situ* zymography revealed an intense activation of MMPs for all groups after acid etching. MMP activity was significantly greater in etched dentin compared with untreated dentin (*p*<0.05), but no difference among acid groups was found (Figure 2).

### Scanning electron microscopy (SEM)

**Figure 3:**
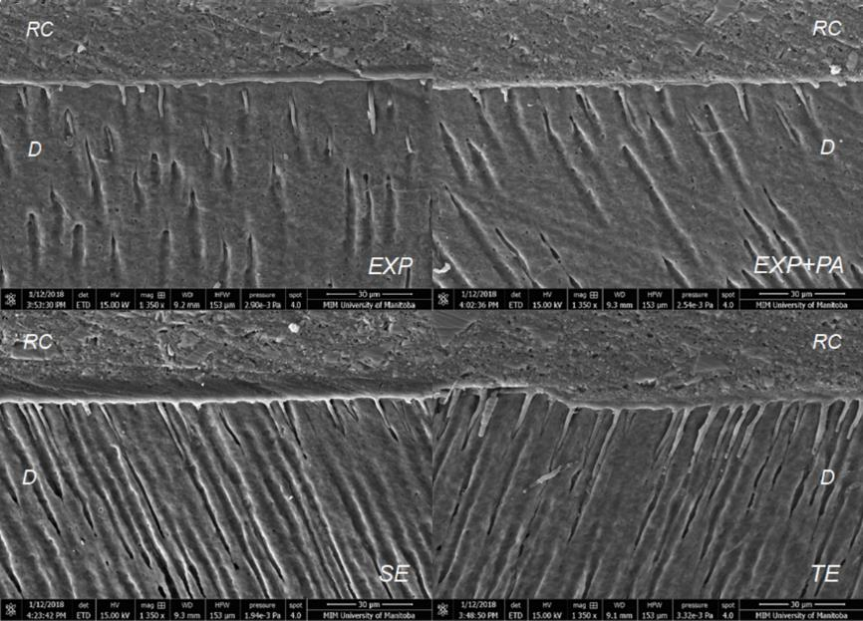
SEM images of the resin-dentin interface with 1350x magnification. EXP - experimental phosphoric acid; EXP+PA - experimental phosphoric acid 10% PA; TE - total-etching system and SE - self-etching system. RC: resin composite; D: dentin.

**Figure 4:**
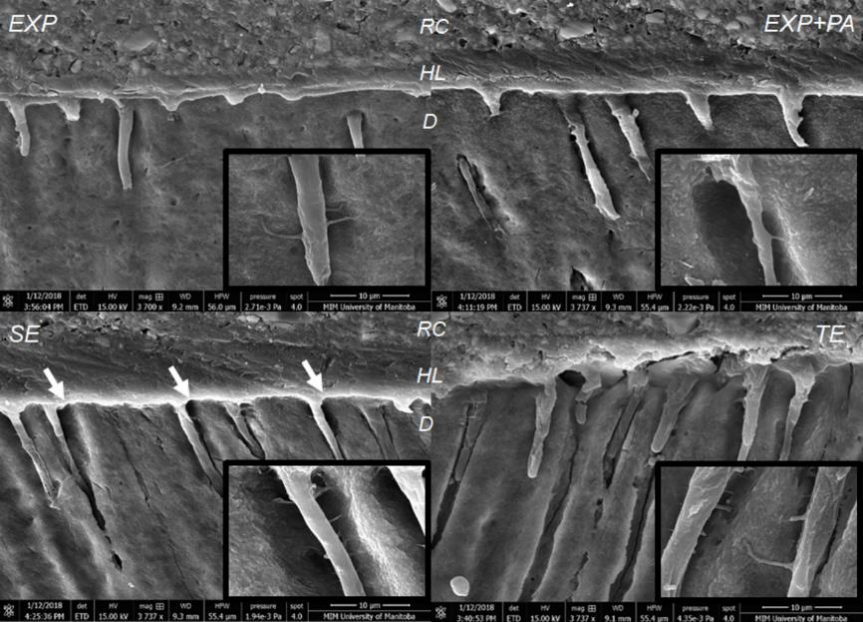
SEM images of the resin-dentin interface with 3737x and 21600x magnification. White arrows point to gaps between resin tags and dentin tubules openings. EXP - experimental phosphoric acid; EXP+PA - experimental phosphoric acid 10% PA; TE - total-etching system and SE - self-etching system. CR: resin composite; HL: hybrid layer.

SEM images revealed the presence of a thick and continuous hybrid layer in all groups (Figures 3 and 4). Resin tags with lateral branches (microtags), indicative of a proper adhesive system penetration in dentinal tubules and microtubules, were also evident for all acid treatments (Figure 4). Reverse cone-shaped resin tags in close contact to dentin walls at the opening of dentinal tubules can be observed for EXP, EXP+PA and TE; gaps between the dentin walls and the resin tags at the tubules’ openings were found only for SE. At deeper portions of the dentinal tubules, EXP+PA and SE presented smaller caliber tags compared with EXP and TE (Figure 4).

## Discussion

The findings of this study indicate that 15 s of application using a PA etchant was not sufficient to significantly prevent the activation of MMPs. Those results can be due to the short application time or the subsequent rinsing of the samples that limits the time for PA to promote MMP inhibition effects. Previous studies used PA biomodification times greater than or equal to 1 min, which are considered clinically unfavorable ^6^ ^,^ ^14^ ^,^ ^15^ ^,^ ^16^. Studies that used PA for periods as short as 15 s incorporated it into the adhesive system or used it as primer, and did not rinse the substrate ^8^ ^,^ ^15^. Nonetheless, recent findings indicate that PA possesses a radical scavenger activity that can impair resin monomers polymerization, resulting in lower bond strength and increased adhesive failures ^5^ ^,^ ^17^ ^,^ ^18^. This property makes PA unsuitable to be incorporated into bond or primer, and rinsing is necessary to remove its residues. PA incorporation into a phosphoric acid formula may be the most suitable for dental purposes, since its collagen crosslinker property remains active even in acid environments ^19^, no extra step is added into the bonding protocol, and PA residues would be rinsed before the application of the primer and/or bond.

The adhesive interface formed with EXP+PA was similar to the ones found on other acid groups. SEM images showed the formation of a continuous uniform hybrid layer with resin tags and microtags in all groups. At deeper portions of the dentinal tubules, however, the formed tags presented a smaller caliber for EXP+PA compared with EXP and TE. These findings are possibly associated to PA’s cross-linker activity, since the aggregation and overlap of collagen fibrils may result in reduced permeability to the adhesive system ^20^ ^,^ ^21^. In addition, PA biomodification has a hydrophobic effect that may also compromise the infiltration of hydrophilic resin monomers of the primer ^(6,21)^. It has been demonstrated that applying PA to collagen films leads to an increase of up to 15° in the surface contact angle, and a consequent decrease in wettability compared to pure collagen films ^21^. Nonetheless, these characteristics did not result in impaired resin-dentin bond strength after 24 h or 6 months of storage. Studies suggest that the most contributing tag features to bonding efficacy may be its shape and attachment to the dentin walls ^22^. In fact, in the present study the SEM images revealed reverse cone-shaped resin tags in close contact with dentin walls at the first section (opening) of dentinal tubules for EXP+PA, EXP and TE, and it was correlated with higher microtensile bond strengths at 24 h compared with SE. The initial portion of the resin tags firmly bonded to dentin walls at the tubules’ openings may have contributed to these results, even when deeper parts of the resin tags presented a smaller caliber.

The findings also indicate that EXP+PA was able to preserve the bonding stable for 6 months and presented higher long-term bond strength compared with experimental and commercial total-etching groups (EXP and TE); therefore, the null hypothesis of the study was rejected. It is interesting to note that bond strength values for SE also remained stable and were not different from EXP+PA after 6 months. MMPs are released and activated by both total- and self-etching systems and can induce progressive degradation of the resin-dentin interface over time ^23^. Nevertheless, since self-etching techniques produce smaller areas of exposed collagen fibrils, a longer period of analysis could be necessary to observe significant interface degradation and reduction in bond strength ^2^ ^,^ ^3^. All groups exhibited a predominance of adhesive failures in the fracture analysis. This is probably due to the reduced size of the beams subjected to microtensile testing, once low forces are required to fracture the adhesive interface of small samples. On the other hand, large specimens may present a higher number of intrinsic defects and, as consequence, can exhibit premature cohesive failures in dentin or resin even under low tensile forces, which prevents the proper assessment of the adhesive bond strength ^24^.

Regarding the mechanical enhancement of the collagen matrix, it has been demonstrated that application times as short as 10 s can improve the collagen`s resistance toward enzymatic breakdown ^8^. Therefore, PA’s cross-linker activity probably promoted the increased bond strength and stability found for EXP+PA, even though significant MMPs inhibition was not achieved. Additionally, recent findings suggest that psychochemical interactions of PA with the collagen matrix may also play a major role on increasing adhesive strength ^25^. PA has bioadhesive properties due to the presence of cathecol moieties, which intermediate the binding of collagen fibrils with the hydrophobic methacrylate adhesives. This property could contribute to create a tight bond between the dentin matrix and the adhesive system that promotes the sealing of the resin-dentin interface ^25^. However, further studies are still needed to determine whether the bioadhesive properties of PA and its cross-linking effects are more relevant than MMP inhibition to the stabilization to the adhesive interface.

In conclusion, the findings indicate that 15 s of application of a phosphoric acid containg 10% PA was not sufficient to inactivate the MMPs at the resin-dentin interface. Nonetheless, dentin biomodification with PA as a natural biocompatible cross-linker incorporated to an etchant formula was able to preserve the resin-dentin bond stability and enhance the long-term bong strength compared with total- and self-etching commercial systems.
